# Dry Wearable Textile Electrodes for Portable Electrical Impedance Tomography

**DOI:** 10.3390/s21206789

**Published:** 2021-10-13

**Authors:** Chang-Lin Hu, I-Cheng Cheng, Chih-Hsien Huang, Yu-Te Liao, Wei-Chieh Lin, Kun-Ju Tsai, Chih-Hsien Chi, Chang-Wen Chen, Chia-Hsi Wu, I-Te Lin, Chien-Ju Li, Chii-Wann Lin

**Affiliations:** 1 Industrial Technology Research Institute, Hsinchu 310, Taiwan; I-Cheng@itri.org.tw (I.-C.C.); timothy_tsai@itri.org.tw (K.-J.T.); ChienJu@itri.org.tw (C.-J.L.); cwlinx@itri.org.tw (C.-W.L.); 2 Department of Electrical Engineering, National Cheng Kung University, Tainan 701, Taiwan; chihhsien_h@mail.ncku.edu.tw (C.-H.H.); n26090423@gs.ncku.edu.tw (C.-H.W.); 3 Department of Electrical and Computer Engineering, National Yang Ming Chiao Tung University, Hsinchu 300, Taiwan; yudoliao@g2.nctu.edu.tw (Y.-T.L.); maoxthexhand.eed04@g2.nctu.edu.tw (I.-T.L.); 4 Division of Critical Care Medicine, Department of Internal Medicine, National Cheng Kung University Hospital, College of Medicine, National Cheng Kung University, Tainan 701, Taiwan; n036961@mail.hosp.ncku.edu.tw (W.-C.L.); cwchen@mail.ncku.edu.tw (C.-W.C.); 5 Department of Emergency Medicine, National Cheng Kung University Hospital, College of Medicine, National Cheng Kung University, Tainan 701, Taiwan; chich@mail.ncku.edu.tw; 6 Department of Biomedical Engineering, National Taiwan University, Taipei 106, Taiwan

**Keywords:** wearable textile electrode, portable electrical impedance tomography, belt, EIT

## Abstract

Electrical impedance tomography (EIT), a noninvasive and radiation-free medical imaging technique, has been used for continuous real-time regional lung aeration. However, adhesive electrodes could cause discomfort and increase the risk of skin injury during prolonged measurement. Additionally, the conductive gel between the electrodes and skin could evaporate in long-term usage and deteriorate the signal quality. To address these issues, in this work, textile electrodes integrated with a clothing belt are proposed to achieve EIT lung imaging along with a custom portable EIT system. The simulation and experimental results have verified the validity of the proposed portable EIT system. Furthermore, the imaging results of using the proposed textile electrodes were compared with commercial electrocardiogram electrodes to evaluate their performance.

## 1. Introduction

Recently, people have paid increasing attention to the prevalence of lung conditions, such as chronic obstructive pulmonary disease, pulmonary fibrosis, and pneumonia [1]. Electrical impedance tomography (EIT) is a noninvasive and radiation-free medical imaging technique providing continuous real-time regional lung aeration [2]. Thus, EIT was regarded as a promising imaging technology for the lung early in the development of EIT systems even though the EIT technique is limited to the low spatial resolution [3]. In 1978, Henderson and Webster used a 2D matrix of 100 electrodes by fixing it on one side of the human chest and a single large electrode on the opposite side of the body to reconstruct a resistivity distribution of the tissues [4]. Brown et al. used 16 electrodes to inject current between adjacent electrodes and then reconstructed the image using a back-projection method [5,6]. To generate lung imaging, the EIT system would inject electrical current with a certain pattern through electrodes attached on the skins of the subjects to apply electrical stimulation [7,8,9]. After that, the conductivity distributions inside their bodies were reconstructed from the voltages collected by electrodes on the body surfaces. Hua et al. calculated the resistivity of the lungs from EIT imaging to examine edema and apnea [10]. The calculated lung volumes could be viewed as an index on lung function to observe lung status from the lung EIT image [11]. In addition, lung EIT imaging has been used to monitor mechanically ventilated patients in intensive care units [12,13].

Additionally, wearable health-monitoring systems are proposed to help people better monitor their health and provide more health-related data to medical personnel [14,15,16,17,18,19]. Thus, several wearable EIT systems have been proposed to monitor lung health [11,20,21,22,23]. Additionally, the electrically conductive textile materials have been developed in various types of clothing for health care. Some properties of textile material are preferred for the realization of these electrodes to provide high comfort for the users while ensuring good quality of measurements, including sufficiently high conductivity, cotton-based fabric, fixed part of the clothes, and standard maintenance (washing and ironing) [24,25]. Therefore, some studies have proposed textile electrodes for electrocardiogram (ECG) measurement devices with high comfort for the users [26,27,28,29]. The textile electrodes belt for EIT has been commercialized by Sentec AG Switzerland [30,31,32]. However, such textile electrodes still require conductive gel, which is placed at the back of the electrodes to reduce skin-contact impedance. Therefore, we created this wearable textile electrode that does not need any conductive gel.

Traditionally, EIT systems assess the internal electrical characteristics of the thorax through attaching adhesive electrodes on the skin of the chest [7]. However, adhesive electrodes could cause discomfort and increase the risk of skin injury in prolonged measurements [33]. Furthermore, the conductive gel between the electrodes and skin could dry up in long-term usage, which could further impact the signal quality [34,35].

To address these problems, in this study, we propose using textile electrodes directly integrated into a clothing belt for EIT lung imaging using our proposed portable EIT system. Our proposed belt containing textile electrodes does not need conductive gel, so it will not have the problem of drying out for long-term usage. Additionally, textile electrodes would make users subjects feel more comfortable compared to traditional rigid electrodes because textile electrodes are soft and flexible.

In this study, we proposed an EIT lung imaging method that uses wearable textile electrodes with our proposed potable EIT system and compared it with the traditional method using commercial ECG electrodes to verify and evaluate the feasibility of this approach.

## 2. Materials and Methods

### 2.1. Portable EIT System

**Figure 1:**
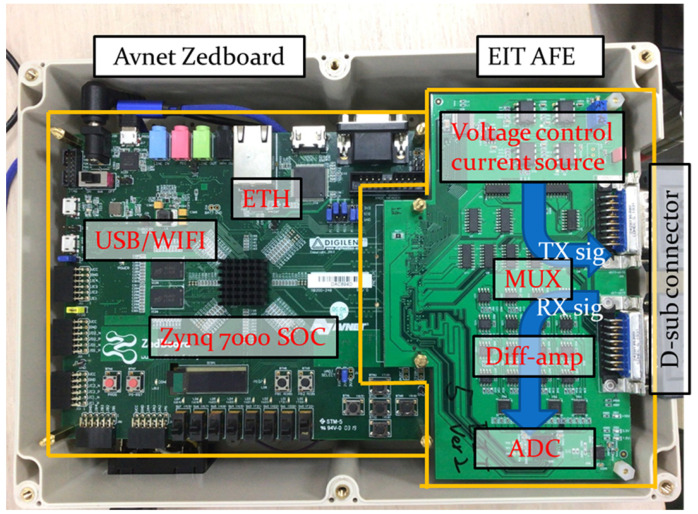
Our proposed portable electrical impedance tomography (EIT) system.

The proposed portable EIT system comprised two main parts: an Avent Zedboard and an EIT analog front-end (AFE) board (Figure 1). The Avent Zedboard is a complete development kit for designers interested in exploring designs using the Xilinx Zynq^®^-7000 All Programmable SoC (xc7z020ffg484-1). In this project, the Avent Zedboard was used to control the EIT AFE and communicate with MATLAB through its JTAG interface.

On the transmitting signal path, the subject under testing is connected to the EIT system’s multiplexers via standard ECG 12-lead measurement cables and electrodes.

The excitation current is generated by the voltage control current source, consisting of a 16-bit digital-to-analog converter, DAC (DAC8820, TI) and a Howland Current Pump circuit. The DAC produces an excitation signal from 0 to +1 V in the frequency range between approximately 25 and 200 kHz. The output from the DAC is converted by the Howland Current Pump circuit to the current range from 0.4 to 10 mA. After that, the multiplexer (DG408, MAXIM) is used to switch the current path to inject the excitation current to the selected electrode.

On the receiving signal path, 16 amplifiers (INA128, TI) detect the difference in voltage between two adjacent channels (Channels 1 and 2, Channels 2 and 3, …, Channels 16 and 1) and amplify the difference by a gain of 3 dB. The outputs of the differential amplifiers are digitized by a 16-channel, 1-volt, and 12-bit analog-to-digital converter ADC (AFE5851, TI) with a sampling rate of 10 Mega samples per second. This ADC device contains 16 variable gain amplifiers amplifying each channel by 1 dB and 16 Anti-Alias Filters, which are third-order digital low-pass filters at 14 MHz. In our proposed EIT system, the image reconstruction frame rate could be up to 30 Hz. In this study, our goal was to verify the feasibility of an EIT lung imaging method that uses wearable textile electrodes with our proposed potable EIT system and compare it with the traditional method using commercial ECG electrodes. Thus, we chose one EIT frame per second to compare the results easily using the two kinds of electrodes from three completed breathing cycles.

### 2.2. Image Reconstruction for Difference EIT

In this study, the proposed portable 16-channel EIT system uses an adjacent current pattern to stimulate electrical signals. The induced voltages were measured between neighboring electrode pairs. A total of 256 recordings were collected each round, where the readings from injected electrodes and their nearest electrodes would be removed. As a result, a 208-voltage dataset would be used to reconstruct the distribution of conductivity [36]. In this study, EIT lung images were reconstructed using the difference imaging method to reflect the volumetric variation of air in the chest during ventilation. In this work, the measurements were recorded twice: one is for the background data with a short exhale, and the other is for the measurement during breathing.

The function for the variations of conductivity could be linearized as follows:

(1) $$\hat{x}=R\left(\lambda \right)\left(v_{m}-v_{b}\right)$$

where $\hat{x}$ represents the changes in conductivity, and R(λ) is the reconstruction matrix depending on the hyperparameter λ. $v_{b}$ is the reference voltage for the background data, and $v_{m}$ is the measured voltage. In this paper, the Electrical Impedance and Diffuse Optical Reconstruction Software (EIDORS) toolbox along with NETGEN and Graz Consensus Reconstruction Algorithm for EIT algorithms were used to reconstruct the EIT imaging [37,38,39].

### 2.3. Experimental Setup and Simulation

**Figure 2:**
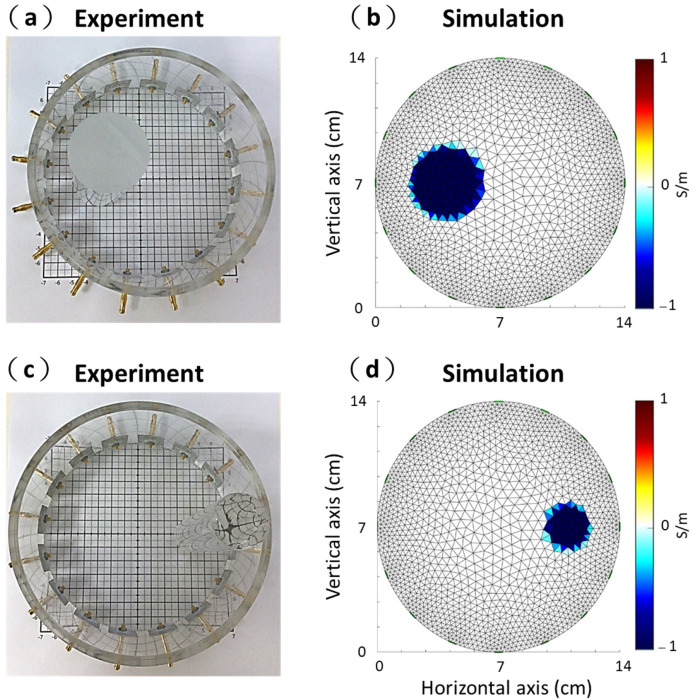
Experimental phantom and simulation setup. (**a**) An acrylic rod with a diameter of 4 cm in the acrylic cylinder. (**b**) A conductivity contrasting inclusion with a diameter of 4 cm using a 2D finite element model. (**c**) An acrylic rod with a diameter of 2.5 cm in the acrylic cylinder. (**d**) A conductivity contrasting inclusion with a diameter of 2.5 cm using a 2D finite element model.

A cylindrical acrylic phantom filled with saline solution was used to demonstrate our portable EIT system validity (Figure 2a,c). The diameter of the phantom was 14 cm with one layer of EIT electrodes mounted radially. One acrylic rod with a diameter of 4 cm was placed on the *x*-axis, −3 cm away from the center of the acrylic phantom (Figure 2a). The other acrylic rod with a diameter of 2.5 cm was placed on the *x*-axis, 4 cm away from the center of the acrylic phantom (Figure 2c). In this study, we reconstructed two kinds of acrylic rods imaging using the difference between the background data (homogeneous medium in the acrylic phantom) and measurement data (two kinds of acrylic rods were placed in the acrylic phantom) (Figure 2a,c). Additionally, a cylinder tank was simulated using 16 electrodes with adjacent patterns using EIDORS [37,40]. Figure 2b,d shows two kinds of circular inclusions with relatively low conductivity in the cylinder tank using a 2D finite element model. The unit for conductivity is Siemens per meter (S/m). The sizes and positions of the EIDORS simulation were the same as those for the experimental setup shown. We evaluated the accuracy of our portable EIT system using performance figures of merit, including the position error (PE) and shape deformation (SD) of the reconstructed images for quantitative analysis [39,41]. PE was defined as the mismatch between the detected position of the images and that of the real object.

(2) $$\mathrm{PE}\;=\left|r_{D}-r_{R}\;\right|$$

where $r_{D}$ represents the detected position of the target images and $r_{R}$ is the real positions of the object. The PE-to-radius ratio (PER) was calculated by dividing the PE by the radius of the object (R) as a metric to evaluate the image quality.

(3) $$\mathrm{PER}\;=\frac{\mathrm{PE}}{R}$$

The SD was calculated by dividing the difference between the detected size of the images and the real size of the object by the real size of the object.

(4) $$\mathrm{SD}=\left|\frac{A_{D}-A_{R}}{A_{R}}\right|$$

where $A_{D}$ represents the detected size of the images and $A_{R}$ is the real size of the object.

### 2.4. Wearable Textile-Electrode EIT Belt

**Figure 3:**
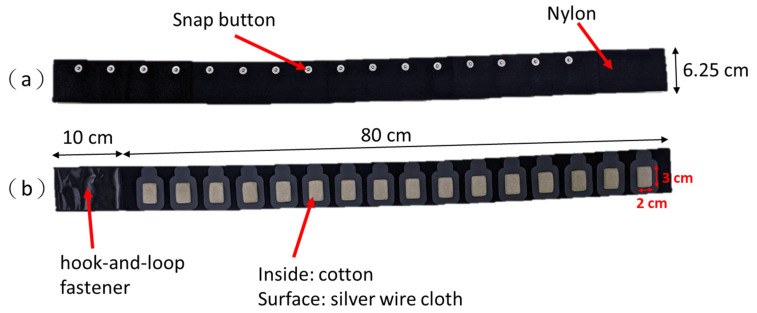
A wearable textile-electrode electrical impedance tomography (EIT) belt. (**a**) The back of the EIT belt with 16 snap buttons. (**b**) The front of the EIT belt with 16 textile electrodes.

We followed these properties of textile material in previous studies to provide high comfort for the users to design our textile-electrode belt [24,25,26,27,28,29]. First, textile material should have high conductivity to provide sensing of the electrical signal with high quality. Second, it should be a cotton-based fabric to provide high comfort on contact fabric and skin. Third, the textile electrode should be a fixed part of the clothes for easy application of the system and position stability of the electrode. Finally, the textile material should allow general maintenance such as washing and ironing. We designed a set of 16 sewn textile electrodes to access EIT data collection to compare to commercial ECG electrodes. Our proposed wearable textile belt for the portable EIT system is shown in Figure 3. Figure 3a presents the back of the EIT belt; the elastic strap was made from nylon with 16 snap buttons stitched on it (Figure 3a). Figure 3b depicts the front of the EIT belt, the 16 textile electrodes were attached to the elastic strap. The inside part of the textile-electrode is cotton, and the surface part connected to the snap button is silver wire cloth. The conductive area of each electrode is 2 × 3 cm. There is a 10 cm hook-and-loop fastener part to fasten the wearable textile-electrode belt (Figure 3b).

The textile electrode having larger surface area than the ECG electrode would have an effect on improving signal quality [42]. Thus, the proposed textile belt without conductive gel still could work in EIT image reconstruction. The material of the textile electrode has passed the ISO 10993-10 test for irritation and skin sensitization. However, skin irritation and sensitization assessment and evaluation in an actual human body using the proposed textile belt require further study.

### 2.5. In Vivo Lung Imaging during Breathing

**Figure 4:**
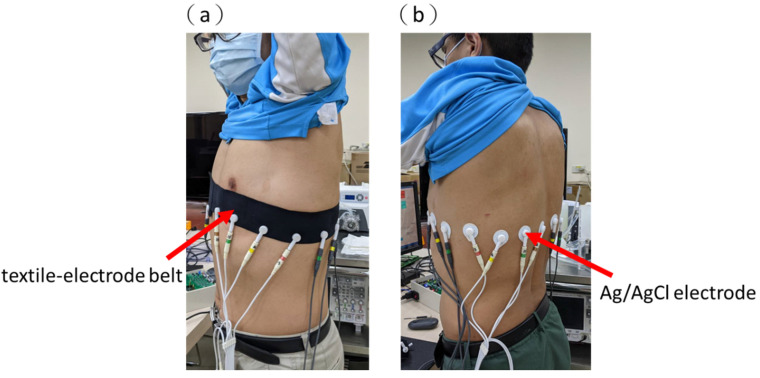
In vivo experiment setup. (**a**) The wearable textile-electrode belt. (**b**) Sixteen commercial electrocardiogram electrodes.

The EIT data of the chest of a healthy volunteer (male, 40 years old) were acquired using our proposed portable EIT system. This experiment was performed according to the World Medical Association Declaration of Helsinki on Ethical Principles for Medical Research Involving Human Subjects [43]. Informed consent was obtained from the healthy volunteer. The in vivo lung EIT images were acquired using the wearable textile-electrode belt (Figure 4a) and compared with those obtained using commercial ECG electrodes (Ag/AgCl electrodes, 30 × 36 mm) (Figure 4b). The wearable textile-electrode belt was put around the chest of the male adult (Figure 4a), and the 16 Ag/AgCl electrodes were tightly attached to chest skin to collect the signal (Figure 4b). In this experiment, information from three completed breathing cycles was recorded. The 27 lung EIT images were acquired using two types of electrodes.

### 2.6. Contact between Surface and Electrodes

The measured voltages are sensitive to the contact impedance between the electrode and skin. The contact impedance of the electrode–skin interface could rise due to the physical postures, movements, and sweat of the patients during EIT operation [44]. Therefore, ensuring that all electrodes make good contact with the chest is crucial for attaining high-quality EIT imaging [45].

**Figure 5:**
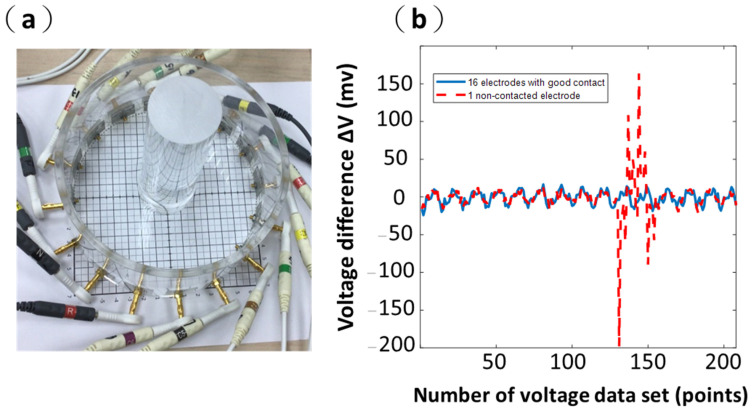
A saline tank is used to calculate the voltage difference ΔV. (**a**) A saline tank with an acrylic rod. (**b**) The voltage difference ΔV with all 16 electrodes having a good contact and 1 non-contacted electrode.

The contact information can be observed by comparing the measurement voltage signals to a background voltage in a defined condition. Thus, we used the difference between the reference voltage and the measurement voltage to determine whether the contact between the skin surface and electrodes is good. The reference voltage refers to background readings. The background data are collected using a saline tank (i.e., homogeneous medium in the acrylic phantom). Then, the background data would be processed to 208 voltages and serve as reference voltages. The measurement data are obtained using a saline tank with an acrylic rod placed inside the acrylic phantom (Figure 5a). Then, the measurement data would be processed to 208 voltages and serve as measurement voltages.

In Figure 5b, the blue line shows that the difference between the reference voltage and the measurement voltage is stable, and the difference ranged from approximately −12.2 to 12.2 mV when all 16 electrodes have good contacts. The red dashed line shows two voltage spikes (about −200 and 150 mV) occurring when there is a non-contacted Electrode 11, shown in Figure 5b. The location of voltage spikes also indicates which electrode has a bad contact to the system, providing a fast diagnosis of the electrode-system attachment. The spikes of a voltage difference (ΔV) in one non-contacted electrode are over ten times larger than the values of the voltage difference in the other well-contacted electrodes (Figure 5b).

**Figure 6:**
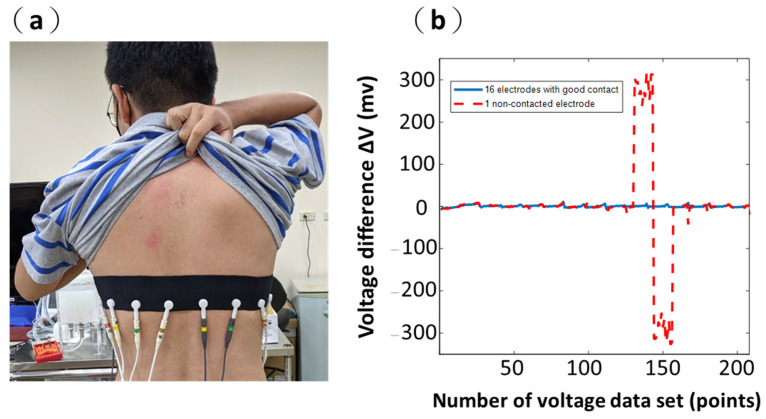
A in vivo is used to calculate the voltage difference ΔV. (**a**) The wearable textile-electrode belt. (**b**) The voltage difference ΔV with all 16 electrodes having a good contact and 1 non-contacted electrode.

In addition, we also used the difference between the reference voltage and measurement voltage using the portable EIT system with a wearable textile-electrode belt to evaluate the contact impedances of the electrode–skin interface. The in vivo lung EIT data were acquired using the wearable textile-electrode belt (Figure 6a). For EIT lung imaging, we ask the subject to fully exhale unnecessary chest movement (to avoid changing the contact condition) to collect the background data. Then, the data would be processed to 208 voltages and serve as reference voltages. The measurement data would be recorded during normal breathing. Then, the measurement data would be processed to 208 voltages and serve as measurement voltages. The measurement voltages during normal breathing would then be compared with the reference voltages and their difference. Finally, this would be used to reconstruct EIT imaging.

In Figure 6b, the blue line shows that the difference between the reference voltage and the measurement voltage is stable, and the difference ranged from approximately −9.76 to 9.76 mV when all 16 electrodes have good contacts. The red dashed line in Figure 6b shows the difference between the two kinds of voltages when there was one non-contact at Electrode 11. In contrast, two spikes’ values in the difference (about −300 and 300 mV) were observed in adjacent electrodes to Electrode 11 with the non-contacted electrode (Figure 6b). The location of voltage spikes also indicates which electrode has a lossy contact to the skin, providing a fast diagnosis of the electrode–body attachment. The values of these spikes in the difference constants are over ten times larger than the values of the difference with the other well-contacted electrodes (Figure 6b). The spikes of the difference caused by bad contact impedance were similar in phantom and in vivo.

A threshold for determining the poor contact condition should be prepared before the system is commercialized. In this article, we have not discussed how to distinguish the contact situations in detail, as a high volume of experiments might be conducted for verification of influence between them and create a reliable standard. In this study, we would like to point out the possibility of using abnormal voltage readings as an indicator of the contact situation using Figure 5b and Figure 6b. A more solid setup would be determined after the system moves to the clinical stage.

## 3. Results and Discussion

### 3.1. Experimental and Simulation Results

**Figure 7:**
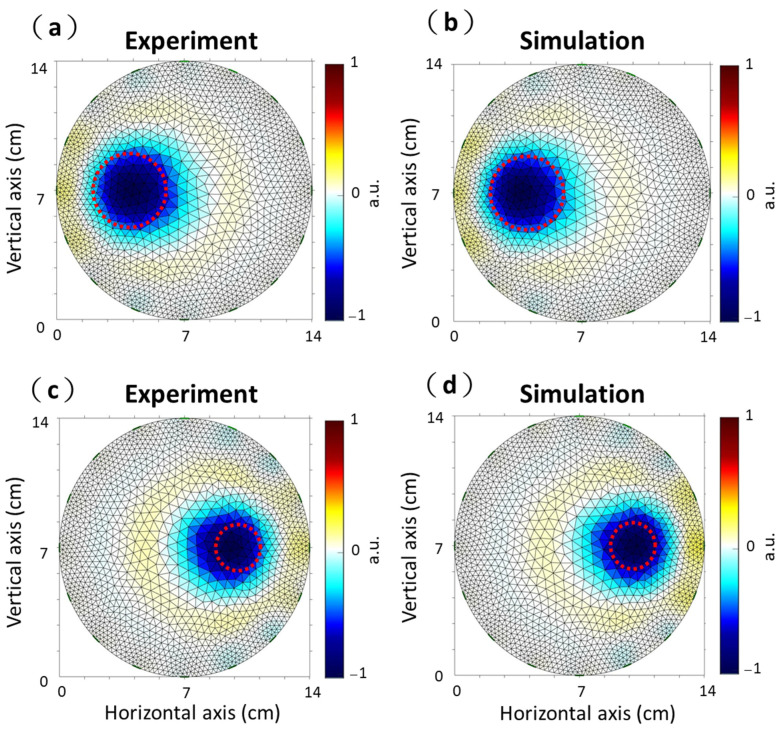
Experimental and simulated electrical impedance tomography (EIT) images. (**a**) An acrylic rod with a diameter of 4 cm in the acrylic cylinder. (**b**) A conductivity contrasting inclusion with a diameter of 4 cm using a 2D finite element model. (**c**) An acrylic rod with a diameter of 2.5 cm in the acrylic cylinder. (**d**) A conductivity contrasting inclusion with a diameter of 2.5 cm using a 2D finite element model.

Figure 7a,c shows the experimental images of the two kinds of acrylic rods with 4 and 2.5 cm diameter using our proposed portable EIT system. Figure 7b,d shows the simulated images of the cylinder tank’s two kinds of circular inclusions. Table 1 lists the SDs, PEs, and PERs values of the EIT images obtained with experimental and simulation results. In the experimental results, the SDs, PEs, and PERs were 16.51%, 1.13 mm, and 5.65% for the diameter of 4 cm and 19.41%, 1.32 mm, and 10.56% for diameter of 2.5 cm. In the simulation results, the SDs, PEs, and PERs were 13.42%, 1.12 mm, and 5.6% for the diameter of 4 cm and 15.1%, 1.02 mm, and 8.16% for the diameter of 2.5 cm. We compared the SDs, PEs, and PERs between the simulation and experimental results. The SDs, PEs, and PERs of the simulation and experimental results were similar. It proves that the experimental imaging from proposed portable EIT system corresponded to the theoretical imaging form the simulation.

### 3.2. Results from Commercial ECG Electrodes (Ag/AgCl Electrodes)

**Figure 8:**
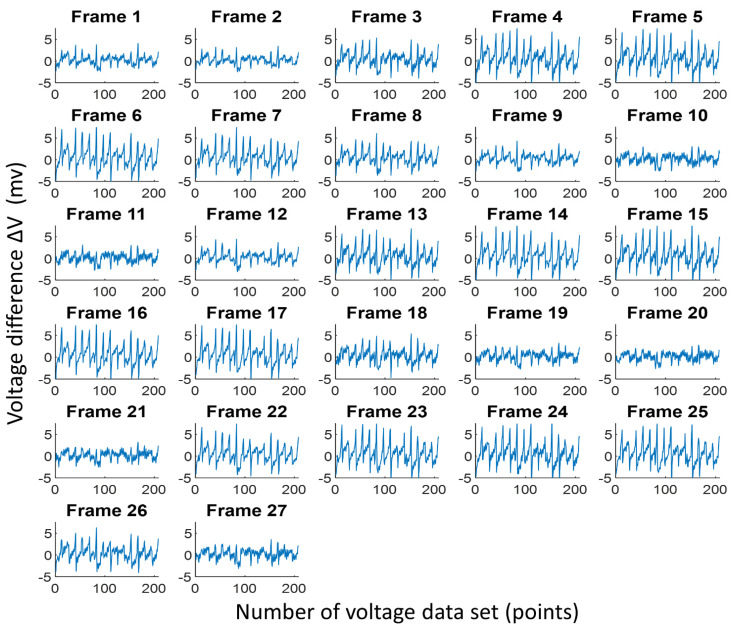
The difference between the reference voltage and measurement voltage from 27 lung electrical impedance tomography data using commercial electrocardiogram electrodes.

Figure 8 shows the difference between the reference voltage and measurement voltage from 27 lung EIT data using commercial ECG electrodes from Frame 1 to Frame 27. The horizontal axis represents the 208-voltage dataset obtained using our proposed portable EIT system, and the vertical axis is the voltage difference ΔV. Here, the unit of voltage difference ΔV is mV. The differences in the 27 lung EIT data were stable, and the differences ranged from −4.88 to 4.88 mV. Thus, the 16 commercial ECG electrodes had good contact with the chest skin.

**Figure 9:**
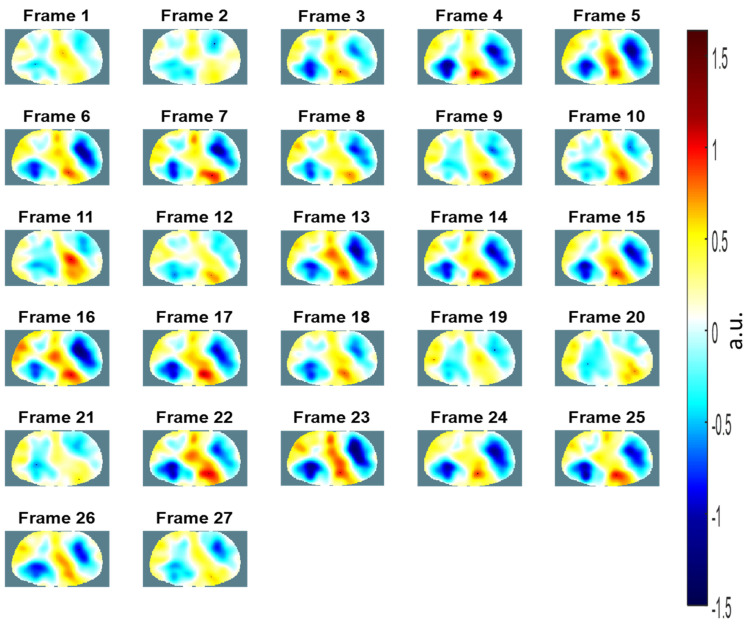
The electrical impedance tomography images of the chest over three breathing cycles using commercial electrocardiogram electrodes.

The EIT images of the chest (i.e., from Frame 1 to Frame 27) over three breathing cycles using a portable EIT system using commercial ECG electrodes are shown in Figure 9. Each EIT image shows resulting conductivity distributions of the chest and consists of a matrix of 129 × 77 pixels (Figure 9). The relative impedance values were calculated to reconstruct EIT images that represent relative changes in ventilation. Therefore, EIT images are commonly expressed in arbitrary units (a.u.) [25,46]. The lung tissue impedance changes by approximately 5% during the cyclic quiet breathing process, and the change could reach up to 300% during deep breathing from residual volume to total lung capacity [47,48]. The increase in air volume during inspiration should increase the impedance of the lung proportionally [49]. Thus, we could assess the distribution of ventilation from the conductivity distributions of the chest. Frame 1 at the top-left corner of the figure represents exhalation, and Frame 5 at the top-right corner of figure represents inhalation (Figure 9). We could find that the voltage differences during inhalation periods from Frame 4 to 8, 13 to 17, and 22 to 26 are larger than those during exhalation periods from Frame 1 to 2, 9 to 12, and 18 to 21, shown in Figure 8 and Figure 9. In addition, we could find that EIT imaging of the left lung is smaller than that of right lung shown in Figure 9. In a study, the functional EIT images could show higher ventilation and perfusion in the right lung than in the left lung [46]. Additionally, the asymmetrical position of the heart could cause asymmetrical EIT imaging between the left and right lungs [50].

**Figure 10:**
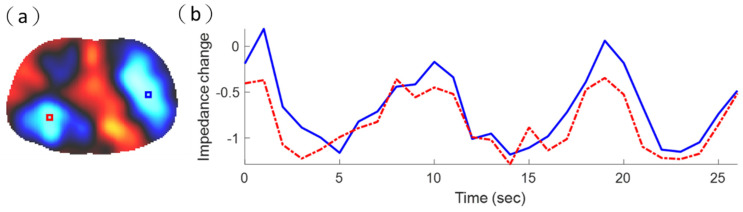
The reconstructed electrical impedance tomography (EIT) image obtained using commercial electrocardiogram electrodes. (**a**) EIT imaging of lung ventilation. (**b**) Two-pixel waveforms in the left lung (red) and right lung (blue).

Figure 10a shows the reconstructed EIT image using commercial ECG electrodes. The impedance changes in pulmonary ventilation were represented in the corresponding pixels using different color tones depending on their magnitude of conductivity. The largest values were shown in bright red, and the smallest values were shown in bright blue. The blue areas in such a functional EIT image represent the ventilated lung regions (Figure 10a) [51]. Figure 10b shows two-pixel waveforms in the left lung (red) and right lung (blue). In Figure 10a, the red pixel waveform represents the tidal variation (TV) of the pixel in the red box. Additionally, the blue pixel waveform represents the TV of the pixel in the blue box. Here, we used TV to measure the distribution of ventilation in the red box and blue box pixels. We calculated the difference between the maximum and minimum values of the pixel waveform to represent TV [52,53]. The TV in the blue box of the right lung was 1.35, and the TV in the red box of the left lung was 0.94.

### 3.3. Results from Wearable Textile-Electrode Belt

**Figure 11:**
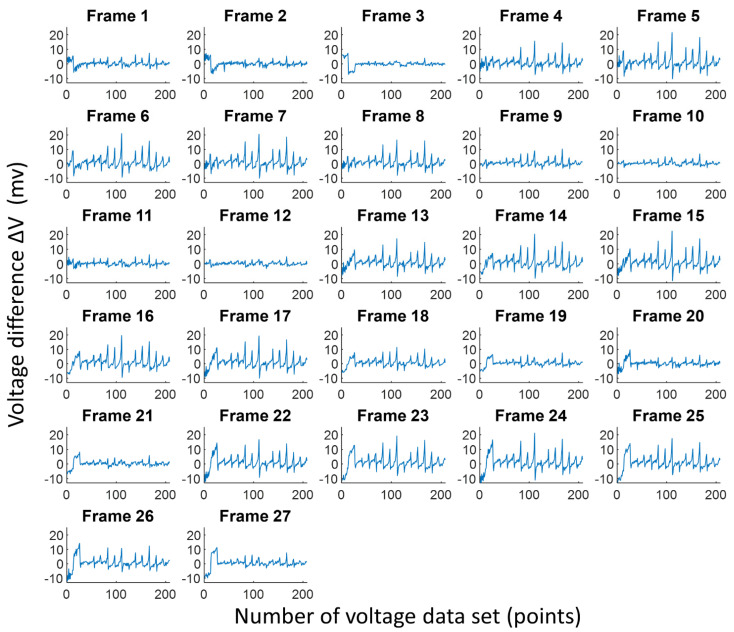
The difference of the reference voltage with the measurement voltage from 27 lung electrical impedance tomography data using a wearable textile-electrode belt.

**Figure 12:**
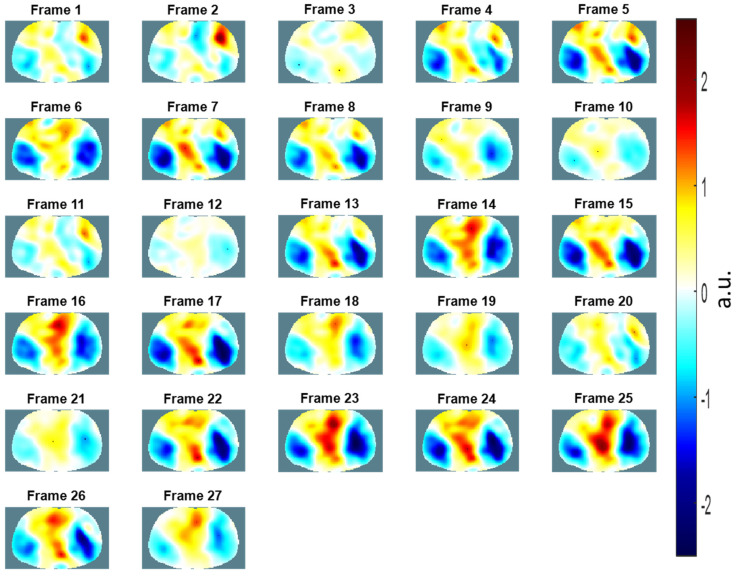
The electrical impedance tomography images of the chest over three breathing cycles using the wearable textile-electrode belt.

Figure 11 shows the difference between the reference voltage and measurement voltage from 27 lung EIT data using our proposed portable EIT system with a wearable textile-electrode belt. The horizontal axis represents the 208-voltage dataset, and the vertical axis is the voltage difference ΔV. The unit of ΔV value is mV. The ΔV values in the 27 lung EIT data were stable, so the 16 textile electrodes had good contact with the chest skin. Additionally, the ΔV ranged from approximately −9.76 to 14.64 mV. Here, voltage difference ΔV represents the conductivity change in the EIT system. We found that the conductivity change using the wearable textile-electrode belt was similar to that obtained using commercial ECG electrodes, as shown in Figure 8 and Figure 11. Thus, using a wearable textile-electrode belt, the reconstructed images are similar to the EIT lung images reconstructed with commercial ECG electrodes as shown in Figure 9 and Figure 12.

The EIT images of the chest (i.e., from Frame 1 to Frame 27) comprised three consecutive breaths using the portable EIT system using the wearable textile-electrode belt (Figure 12). In Figure 12, each EIT image shows the resulting conductivity distributions of the chest and consists of a matrix of 129 × 77 pixels. In Figure 12, Frame 1 at the top-left corner represents exhalation, and Frame 5 at the top-right corner of the figure represents inhalation. It is obvious that the voltage differences during inhalation periods from Frame 4 to 8, 13 to 17, and 22 to 26 are significantly larger than those during exhalation periods from Frame 1 to 3, 9 to 12, and 18 to 21, shown in Figure 11 and Figure 12. The result of the EIT lung images obtained using the wearable textile-electrode belt (Figure 12) was similar to that obtained using commercial ECG electrodes (Figure 9). Thus, our proposed wearable textile-electrode belt could replace commercial ECG electrodes for EIT lung image monitoring.

**Figure 13:**
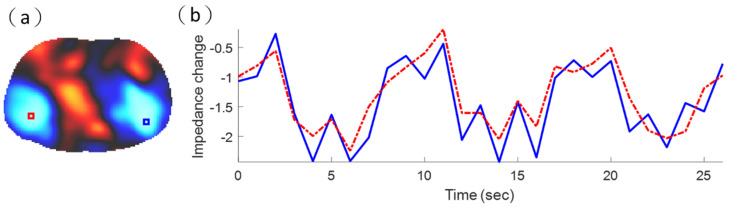
The reconstructed electrical impedance tomography (EIT) image obtained using the wearable textile-electrode belt. (**a**) EIT imaging of lung ventilation. (**b**) Two-pixel waveforms in the left lung (red) and right lung (blue).

Figure 13a shows the reconstructed EIT image obtained using the wearable textile-electrode belt. The largest values were shown in bright red, and the smallest values were shown in bright blue. Figure 13b shows two-pixel waveforms in the left lung (red) and right lung (blue). In Figure 13a, the red pixel waveform represents the TV of the pixel in the red box. Additionally, the blue pixel waveform represents the TV of the pixel in the blue box. The TV in the blue box of the right lung was 2.16, and the TV in the red box of the left lung was 2.05. The results obtained using the wearable textile-electrode belt and commercial ECG electrodes were consistent. The impedance ranges of using the wearable textile-electrode belt (Figure 13) were larger than commercial ECG electrodes (Figure 10). Thus, the wearable textile-electrode belt could provide better discriminability of EIT imaging than commercial ECG electrodes.

## 4. Conclusions

In this study, the simulation and experimental results have verified the validity of our proposed portable EIT system. Furthermore, we proposed using a textile-electrode belt instead of commercial ECG electrodes for EIT lung imaging and evaluated its performance. In this study, the in vivo results have verified the feasibility of our proposed textile-electrode belt. The advantage of the textile-electrode belt is that users would feel more comfortable due to the flexibility and softness of textile electrodes. Additionally, textile electrodes are washable and reusable. Furthermore, our proposed textile-electrode belt could be integrated into clothing.

## Figures and Tables

**Table 1:** Shape deformation (SD), position error (PE), and PE-to-radius ratio (PER) of the EIT images obtained with experiment and simulation, as shown in Figure 7.

	Experiment		Simulation	
	Diameter of 4 cm	Diameter of 2.5 cm	Diameter of 4 cm	Diameter of 2.5 cm
SD (%)	16.51	19.41	13.42	15.1
PE (mm)	1.13	1.32	1.12	1.02
PER (%)	5.65	10.56	5.6	8.16

## Data Availability

Data can be made available upon request.
